# Maternal age at menarche and offspring body mass index in childhood

**DOI:** 10.1186/s12887-019-1659-4

**Published:** 2019-09-04

**Authors:** Hui Wang, Yunting Zhang, Ying Tian, Fei Li, Chonghui Yan, Hui Wang, Zhongchen Luo, Fan Jiang, Jun Zhang

**Affiliations:** 1 MOE-Shanghai Key Laboratory of Children’s Environmental Health, Xin Hua Hospital Affiliated to Shanghai Jiao Tong University School of Medicine, 1665 Kong Jiang Road, Shanghai, 200092 China; 2 Child Health Advocacy Institute, Shanghai Children’s Medical Center Affiliated to Shanghai Jiao Tong University School of Medicine, Shanghai, 200127 China; 3 School of public health, Shanghai Jiao Tong University School of Medicine, Shanghai, 200025 China; 4 Department of Developmental and Behavioral Pediatrics, Shanghai Children’s Medical Center Affiliated to Shanghai Jiao Tong University School of Medicine, 1678 Dong Fang Road, Shanghai, 200127 China

**Keywords:** Early menarche, Body mass index, Intergenerational study

## Abstract

**Background:**

Earlier age of menarche has been associated with an increased risk of chronic diseases during adulthood, but whether early menarche has intergenerational effect is not clear.

**Methods:**

In this population-based cross-sectional study, we recruited children from 26 primary schools using cluster random probability sampling in Shanghai, China, in 2014. We used multiple linear regression models to estimate the adjusted associations of maternal age of menarche (MAM) with offspring body mass index (BMI). We also used the mediation analysis to examine the contribution of maternal BMI and gestational diabetes to offspring BMI.

**Results:**

A total of 17,571 children aged 6–13 years were enrolled, of whom 16,373 had their weight and height measured. Earlier MAM was associated with higher child BMI in boys (− 0.05 z-score per year older MAM, 95% CI − 0.08 to − 0.02) and in girls (− 0.05 z-score per year older MAM, 95% CI − 0.07 to − 0.02). Maternal BMI positively mediated the association of MAM with offspring BMI in both sexes, with mediation effects of 37.7 and 19.4% for boys and girls, respectively.

**Conclusion:**

Early maternal menarche was associated with greater offspring BMI. This study provides evidence for the intergenerational effect in the development of BMI in offspring.

**Electronic supplementary material:**

The online version of this article (10.1186/s12887-019-1659-4) contains supplementary material, which is available to authorized users.

## Background

Menarche marks the onset of reproductive capability in females and the time when resources priority is reallocated from growth to reproduction [1]. Age at menarche has been declining gradually across many developed countries and even more markedly in developing countries in the past several decades [2, 3]. Earlier menarche has been demonstrated to be a risk factor for shorted stature, metabolic syndrome, cardiovascular diseases and polycystic ovarian syndrome in adulthood within one generation [4]. These associations could be explained by the concept of trade-offs between biological functions [5], which suggesting that for a given environment early maturation being a trade-off for additional disease risks in adulthood to maximize reproductive potential [6]. However, whether the pattern of these associations could be extended across generations is unclear. From an evolutionary perspective, exposure during early life not only has long term effects on F1 generation and may also extend to the future generations [7].

Three previous studies from developed countries found that early maternal age of menarche (MAM) was associated with rapid infant growth and childhood obesity in offspring [8–10]. Another study also showed that women with earlier MAM were more likely to have overweight children at 4 to 5 years of age [11]. However, little is known as to the relationship of MAM with offspring BMI beyond preschool stage into childhood in a developing country. Childhood is a critical stage for the establishment of adipose tissue and contributes to the development of adiposity in the later life [12]. Thus, to further examine the intergenerational role of MAM played in childhood body mass index (BMI), we took advantage of a large population-based cross-sectional study, ‘the Shanghai Children’s Health, Education and lifestyle Evaluation (SCHEDULE) study’ to assess the association of MAM with childhood BMI in offspring. We also examined whether these associations varied by sex.

Several studies have found that earlier age of menarche was positively associated with increased risk of gestational diabetes [13–15], which, in turn, may play a role in the development of childhood obesity in offspring [16]. In addition, maternal BMI as a reflection of heritable and shared lifestyle factors could also play a role in offspring BMI [17]. Given, the mechanisms that mediate the associations of MAM with offspring BMI are unclear, we sought to perform mediation analysis to examine the potential contributions of mediators underlying the link between MAM and offspring BMI including maternal BMI, and maternal gestational diabetes.

## Methods

### Data source

**Fig. 1:**
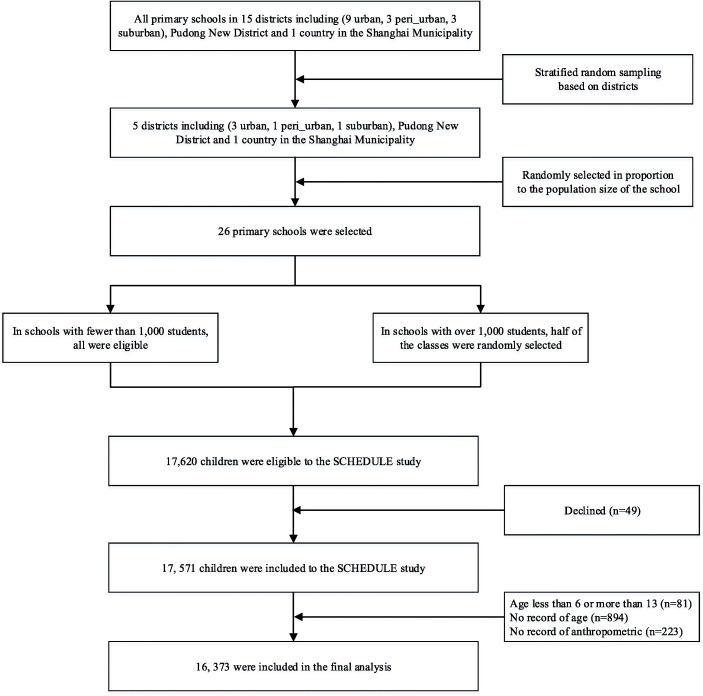
Sampling strategy and recruitment of the SCHEDULE study

The SCHEDULE study is a cross-sectional population-based study which was conducted in Shanghai, China, in June 2014. It was described in detail elsewhere [18, 19]. Briefly, seven districts were randomly selected from the total 19 districts in Shanghai [20]. Among them, 26 primary schools were randomly chosen. All students from Grade one to five (aged from 6 to 13 years) in the chosen schools were eligible for recruitment in this study. For schools with fewer than 1000 students, all of them were eligible, whereas in schools with over 1000 students, only half of the classes were randomly selected. All the students in the selected classes were eligible for this study (as shown in Fig. 1). Sampling weight were computed using inverse probability weighting, which represented the inverse of the combined selection probability for each stage. An invitation letter and a consent form were sent to the parents of the eligible students to inform them of the study and invite them to participate. If the parents agreed to join, they were asked to complete a self-administered questionnaire. Information on parental demographic characteristics (education and family income), perinatal characteristics of the index child (gestational, sex, mode of delivery and birth weight), and offspring characteristics (medical history, food intake frequency, physical activity and mental health during childhood) was collected. Child dietary patterns were reported by parents using a modified food frequency questionnaire (FFQ) with nine food items. Two main factors describing the dietary pattern were derived from the FFQ, including “healthy dietary factor score” and “unhealthy dietary factor score” [21]. The Chinese version of the International Children’s Leisure Activities Study Survey Questionnaire (CLASS-C) was used to measure child time spent in moderate to vigorous physical activity (MVPA) [22]. The time of MVPA was categorized into 3 groups: < 1 h, 1–2 h and ≥ 2 h based on the current guidelines [22]. Physical examination including anthropometric measures (weight, height) and puberty staging was conducted by trained researchers and pediatricians, respectively.

### Exposure

MAM was obtained from the questionnaire reported by the mothers. It was asked as follows: when was your first menstruation? To be consistent with previous studies [8, 9], MAM was recorded in complete year and categorized as ≤11, 12, 13, 14, ≥15. Moreover, to determine if there was a significant linear relationship between MAM and BMI in offspring, we also treated MAM as a continuous variable.

### Mediators

#### Maternal BMI

Based on self-reported height and weight in questionnaire, maternal BMI was calculated as weight (kg) divided by squared height (m^2^).

#### Gestational diabetes

Based on the questionnaire reported by the mothers using the question, “were you diagnosed for gestational diabetes?”, gestational diabetes was categorized as “yes” and “no”.

#### Outcomes

Weight (nearest 100 g) and height (nearest 0.1 cm) were measured by trained staff using a standard protocol [19]. BMI and height were converted into age- and sex-specific z-scores relative to the World Health Organization grow references 5–19 years for comparability with other studies [23].

#### Statistical analysis

Baseline characteristics by MAM were compared using Pearson’s χ^2^ tests and analysis of covariance. Multivariable linear regression was used to examine the adjusted associations of MAM with offspring BMI. We applied sampling weight in the analysis. Whether the associations varied by sex were assessed based on the significance of interaction terms. MAM was also considered as continuous variable in years to assess the linear trends [24]. We select confounders that were potentially associated with both exposure (i.e., MAM) and outcomes (i.e., offspring BMI) [25]. Based on the literature, model 1 adjusted for age. Model 2 additionally adjusted for mode of delivery, maternal education, household income, child activity, pubertal stage, diet pattern and site of school [4].

Maternal BMI and gestational diabetes were considered as potential mediators rather than confounders because they are more likely be on the pathways from MAM to offspring BMI than causes of MAM. We performed the mediation analysis according to the principles of Baron and Kenny: regressing the mediator(s) on the exposure and confounder, and regress the outcome on the exposure and confounders from which we obtained the indirect effect, the direct effect, total effect and the percentage mediated [26]. (These possible pathways were illustrated in directed acyclic graphs (Additional file 1). Mediation effect was identified through the following criteria: 1) the independent variable was a significant predictor of the mediator (if maternal age of menarche (MAM) was significantly associated with maternal body mass index (BMI)/gestational diabetes during adulthood); 2) the independent variable was a significant predictor of the dependent variable (if MAM was significantly associated with childhood BMI in offspring; 3) the mediator was a significant predictor of the dependent variable and the association between the dependent and independent variable was either partially or fully removed if adjustment for mediators (if Maternal BMI/gestational diabetes was significantly associated with childhood BMI in offspring and the association of maternal MAM with childhood BMI in offspring should be attenuated by adjustment for maternal MAM/gestational diabetes).

Multiple imputation was used to account for missing values of exposures and confounders (among 16,452 participants, MAM was imputed for 11.5%, household income for 29.8%, maternal education for 3.4%, maternal BMI for 2.3%, offspring pubertal stage for 1.6%, mode of delivery for 6.5% and maternal gestational diabetes for 5.4%) based on the flexible additive regression model with predictive mean matching incorporating data on the outcomes, mediators, exposures and other covariates potentially associated with MAM [27]. We imputed missing values 10 times and analyzed the 10 complete datasets separately and summarized the results into single estimated beta-coefficients with confidence intervals adjusted for missing data uncertainty [28]. As a sensitivity analysis, we also performed available case analysis.

To evaluate the robustness of the results to potential unmeasured confounding, we calculated E-value using the publicly available online E-value calculator (https://www.hsph.harvard.edu/tyler-vanderweele/tools-and-tutorials/). The E-value is a measure that represents the minimum strength of association that an unmeasured confounder would need to have with both the exposure and the outcome to fully explain the association [29].

Mediation was assessed from a Sobel test using bootstrapped standard error [30]. Data were analyzed using Stata version 13 (Stata Corp, College Station, Texas, USA) and R version 3.2.2 (R development Core Team, Vienna, Austria).

## Results

A total of 17,571 students completed this population-based survey among the 17,620 eligible individuals, with a response rate of 99.7%. Anthropometric measurements were available for 16,373 participants, of whom 1680 (11.1%) had MAM ≤11 years old, 2955 (20.3%) 12 years, 3939 (27.2%) 13 years, 2819 (19.5%) 14 years and 3173 (21.9%) ≥15 years. The mean age of these participants was 9.2 years (ranging from 6 to 13 years) with SD 1.5 years.

Table 1 shows that earlier MAM was associated with a higher level of education and higher household income. Mothers with earlier MAM were more likely to have gestational diabetes. They were also more likely to have babies by cesarean section and higher BMI during adulthood.

**Table 1:** Baseline characteristics by maternal age of menarche from the SCHEDULE study in China

Maternal age of menarche (in complete years), %*							
Characteristics	n	≤11 (1608)	12 (2955)	13 (3939)	14 (2819)	≥ 15 (3173)	*p* value
Sex							
Boys	8180	48.4	48.5	52.4	54.5	61.6	< 0.001
Girls	7057	51.6	51.5	47.6	45.5	38.4	
Low birthweight							0.17
No	15,241	96.2	97.0	96.8	95.9	96.3	
Yes	558	3.8	3.0	3.2	4.1	3.7	
Mode of delivery							< 0.001
Vaginal	7661	41.3	45.8	47.7	51.3	58.5	
Cesarean	7655	58.7	54.2	52.3	48.7	41.5	
Child age (Mean (SE))	16,677	9.3	9.2	9.2	9.3	9.1	< 0.01
Maternal education							< 0.001
Middle school or below	5341	16.9	22.5	27.6	38.1	50.3	
High school	4268	21.2	28.1	26.9	28.9	25.7	
College or above	6219	60.9	49.4	45.5	33.0	24.0	
Household income annually ($ in RMB)							< 0.001
≤ 30,000	1471	8.1	8.4	9.2	13.3	22.3	
30,000-100,000	5122	34.1	42.2	42.7	46.8	49.6	
100,000-300,000	4063	46.1	41.4	40.2	33.1	23.6	
≥ 300,000	834	11.7	8.0	7.8	6.8	4.5	
Maternal gestational diabetes							< 0.001
No	15,093	95.3	97.1	97.2	97.9	98.6	
Yes	395	4.7	2.9	2.8	2.1	1.4	
Maternal BMI (Mean (SE))	16,001	22.3 (3.4)	21.9 (3.2)	21.7 (3.2)	21.7 (3.3)	21.9 (3.7)	< 0.001

* given as % unless indicate

Table 2 presents that earlier MAM was associated with higher BMI z-score during childhood in offspring in boys (− 0.05 z score per year older MAM, 95% CI, − 0.08 to − 0.02) and in girls (− 0.05 z score per year older MAM, 95% CI, − 0.07 to − 0.02) after adjustment for potential confounders. The association of MAM with offspring BMI z-scores did not vary by sex (*P* value for interaction were 0.74).

**Table 2:** Adjusted associations of maternal age of menarche with offspring BMI in the SCHEDULE study in China using multiple imputation

Maternal age of menarche	Boys		Girls	
	Model 1	Model 2	Model 1	Model 2
	*β* (95% CI)	*β* (95% CI)	*β* (95% CI)	*β* (95% CI)
≤11	REF	REF	REF	REF
12	−0.09 (−0.21 to 0.03)	−0.13 (−0.27 to 0.01)	−0.08 (− 0.18 to 0.02)	− 0.06 (− 0.16 to 0.04)
13	− 0.21 (− 0.33 to − 0.10)	− 0.22 (− 0.36 to − 0.09)	− 0.12 (− 0.21 to − 0.03)	− 0.10 (− 0.20 to − 0.01)
14	−0.23 (− 0.34 to − 0.11)	−0.25 (− 0.39 to − 0.10)	−0.15 (− 0.25 to − 0.05)	−0.15 (− 0.25 to − 0.04)
≥15	−0.28 (− 0.39 to − 0.16)	−0.21 (− 0.35 to − 0.08)	−0.21 (− 0.31 to − 0.11)	−0.19 (− 0.30 to − 0.09)
Continuous	−0.07 (− 0.39 to − 0.16)	−0.05 (− 0.08 to − 0.02)	−0.05 (− 0.07 to − 0.03)	−0.05 (− 0.07 to − 0.02)

Model 1 adjusted age; Model 2 additionally adjusted for mode of delivery, maternal education, household income, child activity, pubertal stage, diet pattern and site of school

Table 3 shows that the associations of MAM with BMI z-scores in offspring were partially mediated by maternal adulthood BMI. The association of MAM with BMI z-score in offspring was partially mediated by maternal BMI in both sexes, with mediation effects of 37.7% in boys, and 19.4% in girls. Gestational diabetes did not mediate the association.

**Table 3:** Total, direct, and indirect effects of maternal age of menarche and 95% CI on BMI with the percentages mediated by maternal BMI z-score, and gestational diabetes

	Boys	Girls
Mediators	*β* (95% CI)	*β* (95% CI)
Maternal BMI		
Indirect effect	−0.015 (− 0.021 to − 0.008)	−0.011 (− 0.017 to − 0.006)
Direct effect	−0.031 (− 0.063 to − 0.001)	−0.048 (− 0.077 to − 0.018)
Total effect	−0.046 (− 0.078 to − 0.013)	−0.059 (− 0.088 to − 0.030)
Percentage mediated	37.7%	19.4%
Gestational diabetes		
Indirect effect	−0.001 (− 0.002 to 0.0001)	−0.000 (− 0.001 to 0.0001)
Direct effect	− 0.048 (− 0.083 to − 0.013)	−0.056 (− 0.085 to − 0.026)
Total effect	−0.048 (− 0.081 to − 0.016)	−0.056 (− 0.085 to − 0.027)
Percentage mediated	NA	NA

Models adjusted for age, maternal education, household income, child activity, pubertal stage, diet pattern and site of school

The sensitivity analysis of available case analysis obtained virtually the same results (Additional file 2). The E-values for observed associations were 1.24 and 1.21 in boys and girls, respectively. E-values for the limits of the 95% confidence interval were 1.18, and 1.13, respectively.

## Discussion

In this large, population-representative study, we found that children whose mothers had earlier menarche appeared to have higher BMI during childhood than children born to mothers with later menarche age. These associations did not vary by sex. Our study adds previous evidences by demonstrating an inter-generation effect of maternal early onset of puberty with offspring BMI, which was possibly mediated by maternal BMI.

Our finding is consistent with three previous studies from US, UK and China [8, 9, 11], showing that children whose mothers had menarche earlier than 12 years had taller stature and obesity risks compared to children whose mother had menarche later than 15 years. Our finding is also partly consistent with one study which suggesting that earlier MAM was not associated with BMI during infancy but higher BMI during childhood in offspring, with the association possibly due to cumulative effect from previous stages [10]. This study had lower follow up rate during infancy from birth to 2 years compared to childhood stage, which potentially caused selection bias during this period. Furthermore, BMI might not be a good indicator of adiposity during infancy when body composition changes rapidly as for childhood [31]. We found that maternal BMI in adulthood mediated the relation between maternal early puberty and higher offspring BMI in childhood which were consistent with previous studies [32]. Maternal BMI could be considered as an indicator for intrauterine environment which plays a critical role in childhood growth [33].

In this large population-based study with anthropometric measurements assessed by pediatricians; several limitations still existed. First, MAM was self-reported with a long time interval which might have introduced recall bias. However, age of menarche is a milestone in women’s reproductive life and could be recalled clearly years later [34]. Furthermore, if recall bias has occurred, it was most likely to be non-differential. Such a misclassification usually biases the result towards the null. Second, we do not have other measures than BMI for body composition. BMI may not be a good measure as indicator for adiposity. However, recent studies have shown that BMI during childhood could be considered as the most useful index for predicting obesity in later life [35]. Third, this study is a cross-sectional study. We could not assess the role of MAM on offspring BMI through the life course. However, the stage of early childhood may be a sensitive period, which is a good indicator for adulthood adiposity [36]. Fourth, the age range of the participants was 6–13 years with 85% classified into prepuberty group which decreased the variability of Tanner stage (i.e. they might have a growth spurt if they gone through puberty when the measurement was taken). Fifth, we do not have information on onset of fathers’ puberty. There is no such robust marker for male maturation as menarche in females [37]. However, maternal puberty maturation has similar effect for both males and females, it is possible that paternal rapid puberty maturation might have the same effect. Sixth, imprecisely measured factors might have confounded the observed association. As in other observational studies, the measurement error in self-reported variable is inevitable. Misclassification of gestational diabetes could attenuate our association and bias the results towards the null. Moreover, the observed association could be partly explained by unmeasured or residual confounding. However, in the analyses, we have adjusted for several potential confounders and further calculated the E-values. Based on the E-values, we found that an unmeasured confounder needs to be associated with both MAM and childhood BMI in offspring by the standard effect size of roughly 1.2 to explain away the association, which is unlikely. Seventh, for this study, we used the multistage cluster sampling for the sample representatives and sampling weight used in the analysis for reflecting the survey methodology, which could offset to some extent the bias that existed in this method. Lastly, information on maternal medical conditions was obtained by self-report; no verification via medical records was performed.

The mechanisms underlying the intergeneration association of early MAM with higher BMI during early puberty in offspring are unclear. Several pathways may operate simultaneously. First, both age at menarche and BMI are strong heritable traits from mothers to the next generation [38]. The significant association could be explained by the shared genetic factors such as *LIN28B* and *PXMP3* even though specific genes for these traits are not comprehensively discovered [39]. Researchers have demonstrated that early menarche associated SNPs has also been found to play a role in rapid growth during childhood and early adolescence [40]. Second, MAM could also be considered as a proxy of intrauterine exposure of estrogen [41], i.e. earlier maternal age of menarche might exert long term effect on endogenous estrogen level [5, 42]. No sex-specific differences in these associations of early MAM with BMI in offspring has also emphasized the importance of transgenerational hormonal programming [42]. Animal studies have demonstrated that estrogenic agents could determine preadipocyte differentiation and formation in vitro through upregulation of PPAR-γ [43]. Estrogen exposure in utero has also associated with offspring metabolic disruption including overweight and obesity [44].

## Conclusion

Our study shows that early menarche might have an intergenerational effect on offspring BMI during childhood. More research is needed to better understand the intergenerational effect on offspring BMI, which may offer a new perspective to childhood obesity intervention.

## Additional files

Additional file 1: **Figure S1.** The association between maternal age of menarche and offspring BMI mediated by maternal BMI and gestational diabetes. (DOCX 46 kb)

Additional file 2: **Table S1.** Adjusted associations of maternal age of menarche with BMI z-scores in the SCHEDULE study in China using available case analysis. (DOCX 13 kb)

## Data Availability

The datasets used and/or analyzed during the current study are available from the corresponding author on reasonable request.

## Abbreviations

CI: Confidence intervals
SD: Standard deviations
SDQ: Strengths and Difficulties questionnaire
WHO: World health organization
