# Supplementary material for: Maternal age at menarche and offspring body mass index in childhood
Source: BMC Pediatr. 2019 Sep 4;19:312. doi: 10.1186/s12887-019-1659-4 (PMC6724332; doi:10.1186/s12887-019-1659-4)
Supplement: Supplementary file 1 — Figure S1. The association between maternal age of menarche and offspring BMI mediated by maternal BMI and gestational diabetes. (DOCX 46 kb) [file 12887_2019_1659_MOESM1_ESM.docx]

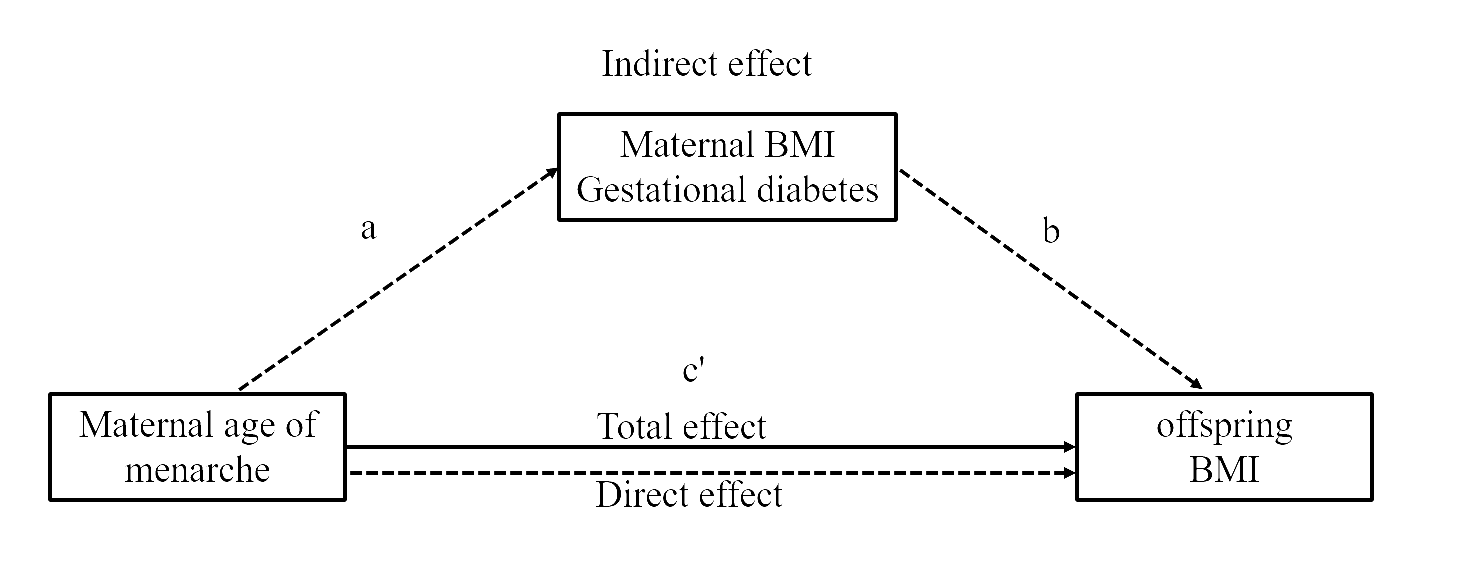


Figure S1 The association between maternal age of menarche and offspring BMI

mediated by maternal BMI and gestational diabetes
