# Supplementary material for: Maternal age at menarche and offspring body mass index in childhood
Source: BMC Pediatr. 2019 Sep 4;19:312. doi: 10.1186/s12887-019-1659-4 (PMC6724332; doi:10.1186/s12887-019-1659-4)
Supplement: Supplementary file 2 — Table S1. Adjusted associations of maternal age of menarche with BMI z-scores in the SCHEDULE study in China using available case analysis. (DOCX 13 kb) [file 12887_2019_1659_MOESM2_ESM.docx]

Table S1 Adjusted associations of maternal age of menarche with BMI z-scores in the SCHEDULE study in China using available case analysis

| Maternal  age of menarche | BMI z-score | |
| --- | --- | --- |
|  | Model 1  β (95% CI) | Model 2  β (95% CI) |
| ≤11 | Reference | Reference |
| 12 | -0.09 (-0.17 to -0.01) | -0.07 (-0.17 to 0.02) |
| 13 | -0.17 (-0.24 to -0.09) | -0.15 (-0.24 to -0.06) |
| 14 | -0.18 (-0.26 to -0.10) | -0.14 (-0.23 to -0.04) |
| ≥ 15 | -0.23 (-0.31 to -0.15) | -0.18 (-0.28 to -0.08) |
| Continuous | -0.03 (-0.05 to -0.02) | -0.03 (-0.05 to -0.01) |

Model 1 sex and age at measurement; Model 2 additionally adjusted mode of delivery, sex, maternal education, household income, child activity, pubertal stage, diet pattern and site of school.
